# Supplementary material for: Accessing the degree of Majorana nonlocality in a quantum dot-optical microcavity system
Source: Sci Rep. 2022 Feb 7;12:1983. doi: 10.1038/s41598-022-05855-y (PMC8821597; doi:10.1038/s41598-022-05855-y)
Supplement: Supplementary file 1 — Supplementary Information. [file 41598_2022_5855_MOESM1_ESM.pdf]

Supplementary information: Accessing the degree of Majorana nonlocality in a quantum dot-optical microcavity system

L. S. Ricco,[1] V. K. Kozin,[1,2] A. C. Seridonio,[3,4] and I. A. Shelykh[1,2]

[1]*Science Institute, University of Iceland, Dunhagi-3, IS-107, Reykjavik, Iceland*

[2]*Department of Physics, ITMO University, St. Petersburg 197101, Russia*

[3]*São Paulo State University (Unesp), School of Engineering, Department of Physics and Chemistry, 15385-000, Ilha Solteira-SP, Brazil*

[4]*São Paulo State University (Unesp), IGCE, Department of Physics, 13506-970, Rio Claro-SP, Brazil*

(Dated: January 25, 2022)

I. CONDUCTION BAND GREEN'S FUNCTION DERIVATION

The quantum dot conduction band Green's function $\boldsymbol{G}^c(\omega) \equiv \langle\langle d_c; d_c^\dagger \rangle\rangle_\omega$ [Eq. (8) in the main text] can be derived through successive applications of the equation-of-motion (EOM) technique [1, 2]. For retarded Green's functions in the spectral domain, the EOM reads

$$(\omega + \imath\delta)\boldsymbol{G}_{A_i,B_j}(\omega) = [A_i, B_j^\dagger]_+ + \langle\langle [A_i, \mathcal{H}]; B_j^\dagger \rangle\rangle_\omega, \tag{1}$$

where $\boldsymbol{G}_{A_i,B_j}(\omega) \equiv \langle\langle A_i; B_j^\dagger \rangle\rangle_\omega$ is the retarded Green's function in the notation adapted from Zubarev [2, 3], $\delta \to 0^+$ is a positive infinitesimal number, A_i and B_j are operators belonging to the Hamiltonian $\mathcal{H}$ of the system [Eq. (1) in the main text] and $[\cdots, \cdots]_+$ is the standard anticommutation relation for fermions [1]. Considering $A_i = B_j = d_c$:

$$(\omega - \omega_c + \imath\delta)\boldsymbol{G}^c(\omega) = 1 + \sqrt{2}\mathcal{V}\sum_{\boldsymbol{k}} \langle\langle c_{\boldsymbol{k},e}; d_c^\dagger \rangle\rangle - \Omega_R \langle\langle d_v c; d_c^\dagger \rangle\rangle_\omega \\ - t_c \langle\langle f; d_c^\dagger \rangle\rangle_\omega - \Delta_c \langle\langle f^\dagger; d_c^\dagger \rangle\rangle_\omega, \tag{2}$$

with $t_c = (\lambda_L - \lambda_R)/\sqrt{2}$, $\Delta_c = (\lambda_L + \lambda_R)/\sqrt{2}$ and $f = (\gamma_L + \imath\gamma_R)/\sqrt{2}$ is a complex fermionic operator built from combination of left (γ_L) and right (γ_R) MZMs [4], and can acquire a nonlocal feature depending on the distance between these 'half-fermionic' Majorana states. As noticed in Eq. (2), the high-order Green's function $\langle\langle d_v c; d_c^\dagger \rangle\rangle_\omega$ arises due to the light-matter coupling term given by $\mathcal{H}_{int}$ [Eq. (2) in the main text]. According to the EOM:

$$(\omega + \imath\delta)\langle\langle d_v c; d_c^\dagger \rangle\rangle_\omega = [d_v c, d_c^\dagger]_+ + \langle\langle [d_v c, \mathcal{H}]; d_c^\dagger \rangle\rangle_\omega = \langle\langle [d_v c, \mathcal{H}]; d_c^\dagger \rangle\rangle_\omega, \tag{3}$$

once $[d_v c, d_c^\dagger]_+ = 0$ and the commutation relation

$$[d_v c, \mathcal{H}] = \omega_0 d_v c + \omega_v d_v c - \Omega_R (d_v d_v^\dagger d_c + c^\dagger c d_c). \tag{4}$$

Thus, Eq. (3) becomes

$$(\omega - \omega_0 - \omega_v + \imath\delta)\langle\langle d_v c; d_c^\dagger \rangle\rangle_\omega = -\Omega_R \langle\langle d_v d_v^\dagger d_c; d_c^\dagger \rangle\rangle_\omega - \Omega_R \langle\langle c^\dagger c d_c; d_c^\dagger \rangle\rangle_\omega, \tag{5}$$

where new high-order Green's functions $\langle\langle d_v d_v^\dagger d_c; d_c^\dagger \rangle\rangle_\omega$ and $\langle\langle c^\dagger c d_c; d_c^\dagger \rangle\rangle_\omega$ arises. In order to find an analytical expression for $\langle\langle d_v c; d_c^\dagger \rangle\rangle_\omega$, we prevent the emergence of more high-order Green's functions in the EOM process by considering the following truncation:

$$\langle\langle d_v d_v^\dagger d_c; d_c^\dagger \rangle\rangle_\omega = \langle d_v d_v^\dagger \rangle \langle\langle d_c; d_c^\dagger \rangle\rangle_\omega = (1 - \langle d_v^\dagger d_v \rangle)\langle\langle d_c; d_c^\dagger \rangle\rangle_\omega \\ = \langle d_c^\dagger d_c \rangle \langle\langle d_c; d_c^\dagger \rangle\rangle_\omega, \tag{6}$$

wherein we considered the one-electron assumption $\langle d_c^\dagger d_c\rangle + \langle d_v^\dagger d_v\rangle = 1$ valid for all times [5] and

$$\langle\langle c^\dagger c d_c; d_c^\dagger\rangle\rangle_\omega = \langle c^\dagger c\rangle\langle\langle d_c; d_c^\dagger\rangle\rangle_\omega. \tag{7}$$

Without loss of generality, in the truncation scheme of Eqs. (6) and (7) we turned the high-order Green's functions of Eq. (5) into the conduction level Green's function $\langle\langle d_c; d_c^\dagger\rangle\rangle_\omega$ modulated by the associated mean number of excitations in the QD $\langle d_c^\dagger d_c\rangle = \langle n_c\rangle$ and the mean number of photons within the optical cavity $\langle c^\dagger c\rangle = N_{ph}$, respectively. Thus, Eq. (5) reads

$$(\omega - \omega_0 - \omega_v + \imath\delta)\langle\langle d_v c; d_c^\dagger\rangle\rangle_\omega = -\Omega_R(\langle n_c\rangle + N_{ph})\langle\langle d_c; d_c^\dagger\rangle\rangle_\omega. \tag{8}$$

Considering that the $\langle n_c\rangle + N_{ph} = \langle\nu\rangle$ gives the mean photon occupation in the cavity, Eq. (8) turns into

$$\langle\langle d_v c; d_c^\dagger\rangle\rangle_\omega = \frac{-\Omega_R\langle\nu\rangle}{(\omega - \omega_0 - \omega_v + \imath\delta)}\langle\langle d_c; d_c^\dagger\rangle\rangle_\omega. \tag{9}$$

Substituting Eq. (9) into (2), we find

$$\begin{aligned}(\omega - \omega_c + \imath\delta)\boldsymbol{G}^c(\omega) = 1 + \sqrt{2}\mathcal{V}\sum_{\boldsymbol{k}}\langle\langle c_{\boldsymbol{k},e}; d_c^\dagger\rangle\rangle + \Sigma_{ph}^c(\omega)\boldsymbol{G}^c(\omega)\\ - t_c\langle\langle f; d_c^\dagger\rangle\rangle_\omega - \Delta_c\langle\langle f^\dagger; d_c^\dagger\rangle\rangle_\omega,\end{aligned} \tag{10}$$

where $\Sigma_{ph}^c(\omega) = \frac{\Omega_R^2\langle\nu\rangle}{\omega-\omega_0-\omega_v+\imath\delta}$ [Eq. (9) in the main text] is the self-energy associated to the valence-to-conduction level transition in the quantum dot induced by the single-mode photonic field of the cavity.

At this point, the Green's function of Eq. (10) that mixes the even conduction operator $c_{\boldsymbol{k},e}$ from the leads with the operator d_c from the dot should be calculated. According to the EOM procedure, this Green's function reads

$$\langle\langle c_{\boldsymbol{k},e}; d_c^\dagger\rangle\rangle_\omega = \frac{\sqrt{2}\mathcal{V}}{\omega + \imath\eta - \epsilon_{\boldsymbol{k}}}\langle\langle d_c; d_c^\dagger\rangle\rangle_\omega \tag{11}$$

Substitution of the expression above into Eq. (10) yields

$$\begin{aligned}(\omega - \omega_c + \imath\delta)\boldsymbol{G}^c(\omega) = 1 + \Sigma_{lead}(\omega)\boldsymbol{G}^c(\omega) + \Sigma_{ph}^c(\omega)\boldsymbol{G}^c(\omega)\\ - t_c\langle\langle f; d_c^\dagger\rangle\rangle_\omega - \Delta_c\langle\langle f^\dagger; d_c^\dagger\rangle\rangle_\omega,\end{aligned} \tag{12}$$

where $\Sigma_{\text{lead}}(\omega) = \sum_{\boldsymbol{k}}\frac{2\mathcal{V}^2}{\omega+\imath\eta-\epsilon_{\boldsymbol{k}}}$ is the self-energy due to the coupling between the QD and the even conduction operators of metallic leads [see Eq.(4) of main text]. In the wide-band limit,

$\mathrm{Re}[\Sigma_{\mathrm{lead}}(\omega)] \rightarrow 0$ and this self-energy is reduced to $-i\Gamma$, which is independent of ω, with $\Gamma = 2\pi\mathcal{V}^2\rho$ [6] as defined in the main text. Thus, Eq. (12) reads

$$(\omega - \omega_c + \imath\delta)\boldsymbol{G}^c(\omega) = 1 - \imath\Gamma\boldsymbol{G}^c(\omega) + \Sigma^c_{ph}(\omega)\boldsymbol{G}^c(\omega) - t_c\langle\langle f; d_c^\dagger\rangle\rangle_\omega - \Delta_c\langle\langle f^\dagger; d_c^\dagger\rangle\rangle_\omega \tag{13}$$

Also through application of EOM [Eq. (1)], the Green's functions of Eq. (10) which mix the operator of the dot conduction level with the fermionic operator built from Majorana modes are given by

$$\langle\langle f; d_c^\dagger\rangle\rangle_\omega = \frac{-t_c\langle\langle d_c; d_c^\dagger\rangle\rangle_\omega + \Delta_c\langle\langle d_c^\dagger; d_c^\dagger\rangle\rangle_\omega}{\omega - \varepsilon_M + \imath\delta} \tag{14}$$

and

$$\langle\langle f^\dagger; d_c^\dagger\rangle\rangle_\omega = \frac{t_c\langle\langle d_c^\dagger; d_c^\dagger\rangle\rangle_\omega - \Delta_c\langle\langle d_c; d_c^\dagger\rangle\rangle_\omega}{\omega + \varepsilon_M + \imath\delta}. \tag{15}$$

Hence, Eq. (13) reads

$$(\omega - \omega_c + i\delta)\boldsymbol{G}^c(\omega) = 1 - \imath\Gamma\boldsymbol{G}^c(\omega) + \Sigma^c_{ph}(\omega)\boldsymbol{G}^c(\omega) + \kappa_1(\omega)\boldsymbol{G}^c(\omega) - t_c\Delta_c\kappa_0(\omega)\langle\langle d_c^\dagger; d_c^\dagger\rangle\rangle_\omega, \tag{16}$$

with

$$\kappa_1(\omega) = \frac{t_c^2}{\omega - \varepsilon_M + \imath\delta} + \frac{\Delta_c^2}{\omega + \varepsilon_M + \imath\delta} \tag{17}$$

and

$$\kappa_0(\omega) = \frac{1}{\omega - \varepsilon_M + \imath\delta} + \frac{1}{\omega + \varepsilon_M + \imath\delta}. \tag{18}$$

In Eq. (16), one can notice the presence of the retarded Green's function $\langle\langle d_c^\dagger; d_c^\dagger\rangle\rangle_\omega$ associated to the superconductivity in the quantum dot induced by the coupling with the Majorana nanowire [7–10] and, according to the EOM technique, is given by

$$(\omega + \omega_c + \imath\delta)\langle\langle d_c^\dagger; d_c^\dagger\rangle\rangle_\omega = -\imath\Gamma\langle\langle d_c^\dagger; d_c^\dagger\rangle\rangle_\omega + \tilde{\Sigma}^c_{ph}(\omega)\langle\langle d_c^\dagger; d_c^\dagger\rangle\rangle_\omega + t_c\langle\langle f^\dagger; d_c^\dagger\rangle\rangle_\omega + \Delta_c\langle\langle f; d_c^\dagger\rangle\rangle_\omega, \tag{19}$$

where $\tilde{\Sigma}^c_{ph}(\omega) = \frac{\Omega_R^2\langle\nu\rangle}{\omega+\omega_0+\omega_\nu+\imath\delta}$ as defined in the main text. Substituting Eqs. (14) and (15) into Eq. (19), we find

$$\langle\langle d_c^\dagger; d_c^\dagger\rangle\rangle_\omega = -t_c\Delta_c K(\omega)\boldsymbol{G}^c(\omega), \tag{20}$$

with

$$K(\omega) = \frac{\kappa_0(\omega)}{\omega + \imath\Gamma + \omega_c - \tilde{\Sigma}^c_{ph}(\omega) - \tilde{\kappa}_1(\omega)} \tag{21}$$

and

$$\tilde{\kappa}_1(\omega) = \frac{t_c^2}{\omega + \varepsilon_M + \imath\delta} + \frac{\Delta_c^2}{\omega - \varepsilon_M + \imath\delta}. \tag{22}$$

Straightforward substitution of Eq. (20) into Eq. (16) yields

$$\boldsymbol{G}^c(\omega) = \boldsymbol{g}_0(\omega) + \boldsymbol{g}_0(\omega)\left[\Sigma_{ph}^c(\omega) + \Sigma_{MZMs}(\omega) - \imath\Gamma\right]\boldsymbol{G}^c(\omega), \tag{23}$$

as the Dyson equation [1, 11] for the quantum dot conduction band, with the self-energy [Eq. (10) in the main text]

$$\Sigma_{MZMs}(\omega) = \kappa_1(\omega) + (t_c\Delta_c)^2\kappa_0(\omega)K(\omega) \tag{24}$$

responsible for renormalizing the dot energy spectrum due to the coupling with the MZMs located at the nanowire. The presence of $\tilde{\Sigma}_{ph}^c(\omega)$ within $K(\omega)$ [Eq. (21)] in the resulting self-energy of Eq. (24) reveals that although the Majorana nanowire is decoupled from the optical cavity, the MZMs are indirectly affected by the photon-induced transitions in the quantum dot.

Finally, by isolating $\boldsymbol{G}^c(\omega)$ in Eq. (23), we can write the expression for the Green's function of the quantum dot conduction band as

$$\boldsymbol{G}^c(\omega) = \frac{\boldsymbol{g}_0(\omega)}{1 + \boldsymbol{g}_0(\omega)\left[\imath\Gamma - \Sigma_{ph}^c(\omega) - \Sigma_{MZMs}(\omega)\right]}, \tag{25}$$

which is the same relation of Eq. (8) in the main text.

II. ANALYSIS OF ANTICROSSING POINTS RELATED TO THE DEGREE OF MAJORANA NONLOCALITY

Following the same procedure of Prada et al. [12], the effective Hamiltonian which describes the QD conduction level coupled to both the left and right MZMs of the Majorana nanowire reads

$$\mathcal{H}^{\text{eff}} = \omega_c d_c^\dagger d_c + \imath\varepsilon_M\gamma_L\gamma_R + \lambda_{c,L}(d_c - d_c^\dagger)\gamma_L + \imath\lambda_{c,R}(d_c + d_c^\dagger)\gamma_R. \tag{26}$$

This Hamiltonian can be rewritten as

$$\mathcal{H}^{\text{eff}} = \frac{1}{2}\psi^\dagger\check{\mathcal{H}}^{\text{eff}}\psi, \tag{27}$$

with $\psi = \begin{pmatrix} d_c & d_c^\dagger & \gamma_L & \gamma_R \end{pmatrix}^T$ and

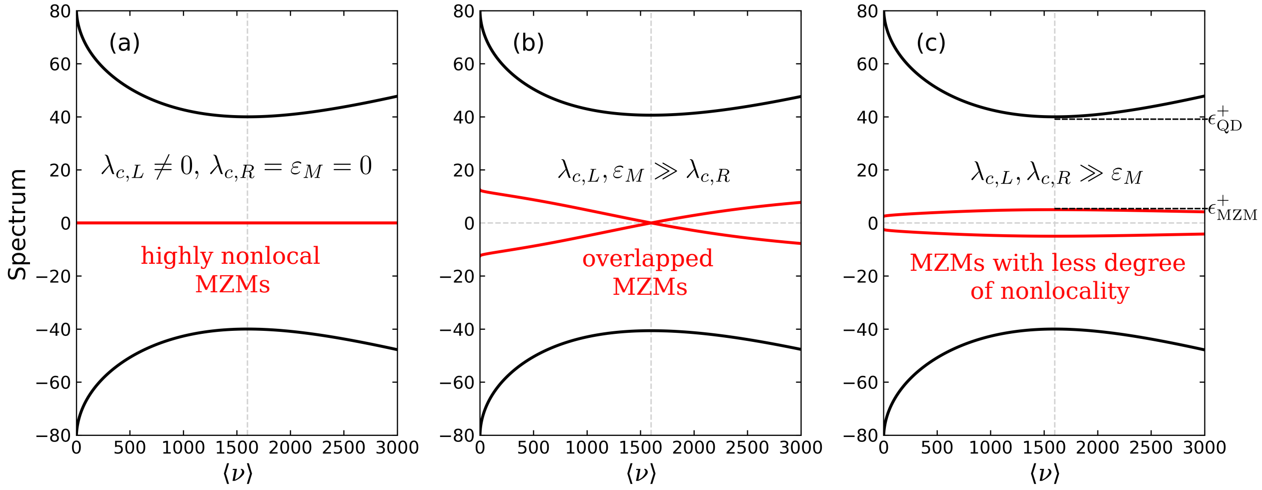

FIG. 1. Low-energy spectrum of the system as a function of mean number of photons $\langle \nu \rangle$, provided by eigenvalues of Eq. (28). The parameters for panels (a), (b) and (c) are the same adopted in Figs. 2(d), 3(d) and 4(d) of the main text, respectively. The gray dashed lines of each panel depicts the points of crossing/anticrossing at $\langle \nu \rangle = (\omega_c/\Omega_R)^2$. For $\omega_c = 100\Gamma$ and $\Omega_R = 2.5\Gamma$, $\langle \nu \rangle = 1600$. The anticrossing points $\epsilon^+_{\rm QD,MZM}$ of panel (c) are given by the analytical expressions of Eqs. (30) and (31).

$$\check{\mathcal{H}}^{\rm eff} = \begin{pmatrix} \omega_c & 0 & -\lambda_{c,L} & \imath\lambda_{c,R} \\ 0 & -\omega_c & \lambda_{c,L} & \imath\lambda_{c,R} \\ -\lambda_{c,L} & \lambda_{c,L} & 0 & \imath\varepsilon_M \\ -\imath\lambda_{c,R} & -\imath\lambda_{c,R} & -\imath\varepsilon_M & 0 \end{pmatrix}. \tag{28}$$

It can be easily noticed that the mean photon occupation $\langle \nu \rangle$ in the microcavity plays no role in Eq. (28). However, it can be seen in Figs. 3(d) and 4(d) of the main text that the crossing/anticrossing points are localized at $\langle \nu \rangle = (\omega_c/\Omega_R)^2$. Thus, the main effect of the cavity photons shown in the gray region of Figs. 2(a), 3(a) and 4(a) can be accounted in $\check{\mathcal{H}}^{\rm eff}$ by considering $\omega_c \to \tilde{\omega}_c = \omega_c - \Omega_R\sqrt{\langle \nu \rangle}$. Although the rightmost peaks of Figs. 2(a), 3(a) and 4(a) are not described by this renormalization, the low-energy spectrum of the system related to the degree of Majorana nonlocality is given by the eigenvalues of the above matrix, which reads

$$E_{\pm(\pm)} = \pm\frac{1}{\sqrt{2}}\left[\sqrt{\left(\frac{(\tilde{\omega}_c^2+\varepsilon_M^2)}{2}+\lambda_L^2+\lambda_R^2\right)(\pm)\sqrt{\left(\frac{\tilde{\omega}_c^2+\varepsilon_M^2}{2}+\lambda_L^2+\lambda_R^2\right)^2-4\left(\frac{\varepsilon_M\tilde{\omega}_c}{2}+\lambda_L\lambda_R\right)^2}}\right]. \tag{29}$$

Fig. 1 shows exactly the low-energy spectrum given by Eq. (29) as a function of the mean number of photons $\langle\nu\rangle$ in the cavity, for the same situations explored in the main text. One can see a perfect matching between Figs. 1 (a), (b) and (c) and Figs. 2(d), 3(d) and 4(d) of the main text, respectively, thus revealing that $\check{\mathcal{H}}^{\text{eff}}$ with the QD conduction level renormalized by the cavity photons $\tilde{\omega}_c$ indeed describes the main behavior of the conductance of the system obtained via Green's functions

As stated above, the anticrossing points are localized at $\langle\nu\rangle = (\omega_c/\Omega_R)^2$, i.e, for $\tilde{\omega}_c = 0$. By applying this condition in the corresponding eigenvalues of the effective Hamiltonian [Eq. (29)], we are able to find the following general analytical expressions for $\epsilon^{\pm}_{\text{QD,MZM}}$:

$$\epsilon^{\pm}_{\text{QD}} = \pm\frac{1}{\sqrt{2}}\sqrt{\left(\frac{\varepsilon_M^2}{2}+\lambda_L^2+\lambda_R^2\right)+\sqrt{\left(\frac{\varepsilon_M^2}{2}+\lambda_L^2+\lambda_R^2\right)^2-4\lambda_L^2\lambda_R^2}} \tag{30}$$

and

$$\epsilon^{\pm}_{\text{MZM}} = \pm\frac{1}{\sqrt{2}}\sqrt{\left(\frac{\varepsilon_M^2}{2}+\lambda_L^2+\lambda_R^2\right)-\sqrt{\left(\frac{\varepsilon_M^2}{2}+\lambda_L^2+\lambda_R^2\right)^2-4\lambda_L^2\lambda_R^2}}. \tag{31}$$

For the case of MZMs with less degree of Majorana nonlocality, corresponding to Fig. 1(c) and Fig. 4(d) of the manuscript, $\lambda_L, \lambda_R \gg \varepsilon_M$, and hence, from Eqs. (30) and (31) we obtain $\Omega_M = \sqrt{\epsilon^{\pm}_{\text{MZM}}/\epsilon^{\pm}_{\text{QD}}} = \sqrt{\lambda_R/\lambda_L}$ for the degree of Majorana nonlocality, which is exactly the same relation originally proposed by Prada et al [12].

[1] H. Bruus and K. Flensberg, *Many-Body Quantum Theory in Condensed Matter Physics: An Introduction*, Oxford Graduate Texts (Oxford University Press, 2004).

[2] D. N. Zubarev, Double-time green functions in statistical physics, Soviet Physics Uspekhi **3**, 320 (1960).

[3] M. M. Odashima, B. G. Prado, and E. Vernek, Pedagogical introduction to equilibrium Green's

functions: condensed-matter examples with numerical implementations, Rev. Bras. Ensino Fís **39**, 10.1590/1806-9126-rbef-2016-0087 (2017).

[4] R. Aguado, Majorana quasiparticles in condensed matter, Riv. Nuovo Cimento **40**, 523 (2017).

[5] B. W. Shore and P. L. Knight, The Jaynes-Cummings Model, Journal of Modern Optics **40**, 1195 (1993), https://doi.org/10.1080/09500349314551321.

[6] P. W. Anderson, Localized magnetic states in metals, Phys. Rev. **124**, 41 (1961).

[7] D. E. Liu and H. U. Baranger, Detecting a majorana-fermion zero mode using a quantum dot, Phys. Rev. B **84**, 201308 (2011).

[8] E. Vernek, P. H. Penteado, A. C. Seridonio, and J. C. Egues, Subtle leakage of a majorana mode into a quantum dot, Phys. Rev. B **89**, 165314 (2014).

[9] L. S. Ricco, M. de Souza, M. S. Figueira, I. A. Shelykh, and A. C. Seridonio, Spin-dependent zero-bias peak in a hybrid nanowire-quantum dot system: Distinguishing isolated majorana fermions from andreev bound states, Phys. Rev. B **99**, 155159 (2019).

[10] L. S. Ricco, V. L. Campo, I. A. Shelykh, and A. C. Seridonio, Majorana oscillations modulated by fano interference and degree of nonlocality in a topological superconducting-nanowire–quantum-dot system, Phys. Rev. B **98**, 075142 (2018).

[11] F. J. Dyson, The *s* matrix in quantum electrodynamics, Phys. Rev. **75**, 1736 (1949).

[12] E. Prada, R. Aguado, and P. San-Jose, Measuring majorana nonlocality and spin structure with a quantum dot, Phys. Rev. B **96**, 085418 (2017).
